# Supplementary material for: Estimating individual treatment effect on disability progression in multiple sclerosis using deep learning
Source: Nat Commun. 2022 Sep 26;13:5645. doi: 10.1038/s41467-022-33269-x (PMC9512913; doi:10.1038/s41467-022-33269-x)
Supplement: Supplementary file 3 — Reporting Summary [file 41467_2022_33269_MOESM3_ESM.pdf]

nature portfolio

Corresponding author(s): Jean-Pierre R. Falet

Last updated by author(s): Jul 19, 2022

Reporting Summary

Nature Portfolio wishes to improve the reproducibility of the work that we publish. This form provides structure for consistency and transparency in reporting. For further information on Nature Portfolio policies, see our Editorial Policies and the Editorial Policy Checklist.

Statistics

For all statistical analyses, confirm that the following items are present in the figure legend, table legend, main text, or Methods section.

n/a	Confirmed	
☐	☒	The exact sample size (*n*) for each experimental group/condition, given as a discrete number and unit of measurement
☐	☒	A statement on whether measurements were taken from distinct samples or whether the same sample was measured repeatedly
☐	☒	The statistical test(s) used AND whether they are one- or two-sided<br>*Only common tests should be described solely by name; describe more complex techniques in the Methods section.*
☐	☒	A description of all covariates tested
☐	☒	A description of any assumptions or corrections, such as tests of normality and adjustment for multiple comparisons
☐	☒	A full description of the statistical parameters including central tendency (e.g. means) or other basic estimates (e.g. regression coefficient) AND variation (e.g. standard deviation) or associated estimates of uncertainty (e.g. confidence intervals)
☐	☒	For null hypothesis testing, the test statistic (e.g. *F*, *t*, *r*) with confidence intervals, effect sizes, degrees of freedom and *P* value noted<br>*Give P values as exact values whenever suitable.*
☒	☐	For Bayesian analysis, information on the choice of priors and Markov chain Monte Carlo settings
☒	☐	For hierarchical and complex designs, identification of the appropriate level for tests and full reporting of outcomes
☒	☐	Estimates of effect sizes (e.g. Cohen's *d*, Pearson's *r*), indicating how they were calculated

Our web collection on statistics for biologists contains articles on many of the points above.

Software and code

Policy information about availability of computer code

Data collection	No software was used.
Data analysis	All experiments were implemented in Python 3.8. Multilayer perceptrons were implemented using the Pytorch library (version 1.7.1). Scikit-Learn (version 0.24.2) was used for the implementation of ridge regression, while Lifelines (version 0.27.0) was used for the Cox Proportional Hazards models.

For manuscripts utilizing custom algorithms or software that are central to the research but not yet described in published literature, software must be made available to editors and reviewers. We strongly encourage code deposition in a community repository (e.g. GitHub). See the Nature Portfolio guidelines for submitting code & software for further information.

Data

Policy information about availability of data

All manuscripts must include a data availability statement. This statement should provide the following information, where applicable:

- Accession codes, unique identifiers, or web links for publicly available datasets
- A description of any restrictions on data availability
- For clinical datasets or third party data, please ensure that the statement adheres to our policy

Data used in this work are controlled by pharmaceutical companies and therefore are not publicly available. Access requests should be forwarded to data controllers.

Human research participants

Policy information about studies involving human research participants and Sex and Gender in Research.

Reporting on sex and gender	Sex was recorded for all study participant, while gender was not. Sex was used as input to our predictive model. A separate analysis of model performance for male and female sex was conducted and differences in performance were explicitly stated in our Results.
Population characteristics	The following characteristics were recorded as part of the original randomized clinical trials cited in our manuscript and used as input for our model: age, sex, height, weight, time from symptom onset, Expanded Disability Status Scale, Functional Systems Scores, 9-hole peg test, timed 25-foot walk, T2 lesion volume, gadolinium-enhancing lesion count, and normalized brain volume.
Recruitment	All participants were recruited as part of the randomized clinical trials cited in our manuscript. We refer the reader to the trial publications for details about the recruitment process.
Ethics oversight	The study protocol was originally approved by the McGill University Health Center's Research Ethics Board - Neurosciences-Psychiatry (IRB00010120) and then transferred and approved by the McGill University Faculty of Medicine and Health Sciences Institutional Review Board (A03-M14-22A).

Note that full information on the approval of the study protocol must also be provided in the manuscript.

Field-specific reporting

Please select the one below that is the best fit for your research. If you are not sure, read the appropriate sections before making your selection.

[x] Life sciences [ ] Behavioural & social sciences [ ] Ecological, evolutionary & environmental sciences

For a reference copy of the document with all sections, see nature.com/documents/nr-reporting-summary-flat.pdf

Life sciences study design

All studies must disclose on these points even when the disclosure is negative.

Sample size	Sample size estimates were calculated in each clinical trial. For the purpose of our study, the number of clinical trials (and therefore the total sample size of our pooled dataset) was not determined based on a power calculation, since the purpose of our study is not to detect a treatment effect in a previously studied population but rather a predicted sub-population for which expected effect sizes are unknown. To maximize sample size, we combined data from all available phase 3 clinical trials involving anti-CD20 monoclonal antibodies, as well as the only clinical trial to have studied laquinimod in primary progressive multiple sclerosis (MS). We supplemented this dataset with data from all the studies we have access to that investigated either an anti-CD20 antibody or laquinimod in the relapsing-remitting MS population.
Data exclusions	We excluded participants who were observed for less than 6 months, or who were missing one of the 19 input features at the baseline visit. These exclusion criteria were predetermined.
Replication	Generalization error estimates and quantitative findings regarding the characteristics differentiating responders from non-responders were replicated on two separate held-out samples: one sample was a subset from the ORATORIO and OLYMPUS clinical trials, and another was a subset of the ARPEGGIO clinical trial. Both of these attempts were successful attempts at replication. We did not have further attempts at replication.
Randomization	Participants were randomized according to the original clinical trial protocols.
Blinding	Investigators were blinded according to the original clinical trial protocols.

Reporting for specific materials, systems and methods

We require information from authors about some types of materials, experimental systems and methods used in many studies. Here, indicate whether each material, system or method listed is relevant to your study. If you are not sure if a list item applies to your research, read the appropriate section before selecting a response.

Materials & experimental systems

n/a	Involved in the study
☒	☐ Antibodies
☒	☐ Eukaryotic cell lines
☒	☐ Palaeontology and archaeology
☒	☐ Animals and other organisms
☒	☐ Clinical data
☒	☐ Dual use research of concern

Methods

n/a	Involved in the study
☒	☐ ChIP-seq
☒	☐ Flow cytometry
☒	☐ MRI-based neuroimaging
